# Quantifying donor-to-donor variation in macrophage responses to the human fungal pathogen *Cryptococcus neoformans*

**DOI:** 10.1371/journal.pone.0194615

**Published:** 2018-03-29

**Authors:** Mariam Garelnabi, Leanne M. Taylor-Smith, Ewa Bielska, Rebecca A. Hall, Daniel Stones, Robin C. May

**Affiliations:** Institute of Microbiology & Infection and the School of Biosciences, University of Birmingham, Edgbaston, Birmingham, United Kingdom; University of Minnesota, UNITED STATES

## Abstract

Cryptococcosis remains the leading cause of fungal meningitis worldwide, caused primarily by the pathogen *Cryptococcus neoformans*. Symptomatic cryptococcal infections typically affect immunocompromised patients. However, environmental exposure to cryptococcal spores is ubiquitous and most healthy individuals are thought to harbor infections from early childhood onwards that are either resolved, or become latent. Since macrophages are a key host cell for cryptococcal infection, we sought to quantify the extent of individual variation in this early phagocyte response within a small cohort of healthy volunteers with no reported immunocompromising conditions. We show that rates of both intracellular fungal proliferation and non-lytic expulsion (vomocytosis) are remarkably variable between individuals. However, we demonstrate that neither gender, *in vitro* host inflammatory cytokine profiles, nor polymorphisms in several key immune genes are responsible for this variation. Thus the data we present serve to quantify the natural variation in macrophage responses to this important human pathogen and will hopefully provide a useful “benchmark” for the research community.

## Introduction

Cryptococcosis is the leading cause of fungal meningitis worldwide, with the vast majority of clinical cases being caused by *Cryptococcus neoformans*. Pulmonary infection may begin when yeast propagules are inhaled from the environment and engulfed by phagocytic cells patrolling the lung epithelium. Most isolates of cryptococci are remarkably resistant to macrophage killing, and are able to adapt and survive within these myeloid cells causing latent infections that may later disseminate upon immune suppression to cause cryptococcal meningoencephalitis (CM) [1].

The last two decades have seen a decline in HIV-associated CM in developed countries due to improved access to a combination of antiretroviral therapies and effective antifungal treatments [2]. It has been estimated that 20% of global CM cases afflict non-HIV patients [3], with more recent reports indicating a growing concern over CM due to *C*. *neoformans* in otherwise healthy individuals [2–4]. Despite the global distribution of this species, the highest frequency of CM in immunocompetent individuals appears to come from South East Asia, with mortality rates in China, Taiwan and Japan estimated to be at 17%, 70% and 35%, respectively [5–7]. In the United States, recent epidemiological studies suggest the rate of cryptococcal infection among non-HIV patients has risen significantly to match that in HIV-infected individuals [2, 8]. Whilst time to presentation following initial symptoms of infection does not differ between HIV positive and negative patients [4], one of the issues restricting effective disease management is that non-HIV CM patients represent a highly heterogenous group. In a retrospective study that assessed predictors of disease mortality in different groups of cryptococcosis patients, it was shown that it took an average of 68 days to diagnose otherwise healthy individuals with cryptococcosis, while HIV patients were diagnosed within 22 days [9].

This may be due to differences in presentation of symptoms e.g. CM patients with HIV are more likely to present with fever whilst the non-HIV group more frequently presents with abnormal mental status, lung involvement and lesions in the central nervous system (CNS) [4] or due to clinician awareness of cryptococcosis as a complication of HIV. Other studies have shown that tools used to conventionally diagnose cryptococcosis differ in their specificity and accuracy in detecting fungal load between HIV-patients and their healthy counterparts [10].

Treatment regimens for HIV-negative cryptococcosis patients are primarily based on data from studies on HIV patients, due to the scarcity of data within a more relevant context [11]. Hence, an understanding of the host-pathogen interactions at a cellular level that enable ‘otherwise healthy’ individuals to overcome cryptococcal disease would allow healthcare providers more robust treatment strategies in this group, and ultimately improve survival rates.

Engulfed cryptococci are able to manipulate host phagosome maturation, enabling the yeast’s intracellular survival [12, 13]. An interesting feature of *Cryptococcus’s* intracellular parasitism is its ability to undergo vomocytosis (non-lytic expulsion) from the host macrophage either immediately or a few hours after engulfment [14, 15]. This rapid process has been suggested to facilitate cryptococcal dissemination throughout the host whilst evading immune detection, ultimately resulting in passage into the CNS via the Trojan horse model [16, 17].

Macrophages represent a key target cell for *C*. *neoformans*. Mice with depleted macrophages infected with this pathogen showed enhanced survival over their wild type couterparts and significantly lower CNS involvement [18]. Given that both latent and symptomatic infections likely arise from an early intracellular, macrophage-associated, fungal colonization, a key question is how variable this early macrophage response is to the fungus. Here we present quantitative data from the *in vitro* challenge of monocyte-derived macrophages (MDMs) from 15 healthy individuals with *C*. *neoformans* var. *grubii*. Despite the relatively small cohort, there is remarkable variation in macrophage ability to control both intracellular parasitism and vomocytosis of *C*. *neoformans*. This variation is not easily explained either by variation in cytokine signaling or genetic polymorphisms in several immune genes previously implicated in the macrophage response to cryptococci and thus likely reflects either cryptic genetic variability between donors or variation in the local environment experienced by the monocyte prior to *in vitro* differentiation.

## Materials and methods

### *Cryptococcus* strains

*C*. *neoformans var*. *grubii* serotype A, strain KN99α [19] was used in all macrophage challenge experiments. In order to obtain a fluorescently labeled strain suitable for imaging, the wild type strain was biolistically transformed [20, 21] with a plasmid pAG32_GFP [20] encoding for a green fluorescent protein (GFP) and subsequently validated for growth at 37°C and 5% CO_2_ and for sensitivity against several stress conditions mimicking the hostile environment inside phagocytes (S1b and S1c Fig). After 24 hours of growth, serial dilutions of cells were plated onto YPD plates and colony-forming units (CFU) counted. CFUs relative to time point 0, before stress treatment, were calculated.

In addition, GFP expressing transformants were tested for their survival and intracellular proliferation rates (IPR) inside J774 macrophages. For IPR, 0.5 x 10^5^ J774 cells were infected at an MOI 1:10 with either wild type KN99α or GFP-expressing transformants (0.5 x 10^6^ cells/ml) opsonized with 18B7 antibody (a kind gift of Arturo Casadevall) as described previously [20, 22]. The IPRs were assessed after initial 2 and following 24 hours of infection (S1a Fig).

### Donor randomisation

This study was approved by the Science, Technology, Engineering and Mathematics Ethical Review Committee of the University of Birmingham. Under ERN_15–0804, 30-60ml venous blood samples were collected in lithium heparin VACUTAINER^®^ tubes obtained from healthy volunteers with full informed consent, and randomized immediately after donation. Due to unavailabity for repeat blood donations, RG008 was excluded from the study.

### Serum collection

VACUTAINER^®^ tubes with silica bead clot activator (CAT) were used to collect serum during each blood donation, and incubated at 37°C, 5% CO_2_ for one hour prior to centrifugation at 800 x *g* for 10 minutes. Live serum aliquots were thereafter stored at -80°C for cytokine profiling.

### Monocyte isolation, differentiation and culture

Primary peripheral blood monocytes (PBMCs) were isolated from fifteen healthy volunteers by double gradient centrifugation using Percoll^®^ (Sigma-Aldrich, H4522). The dual gradient was created by layering 6 ml of 1.098 g/ml Percoll^®^ (70.9% Percoll^®^, 19.1% H_2_O, 10% 1.5M NaCl) underneath 1.079 g/ml Percoll^®^ (56.3% Percoll^®^, 33.7% H_2_O, 10% 1.5M NaCl); onto which 6 ml of undiluted donor blood was layered on top of, and centrifuged for 8 minutes at 150 x *g*, followed by 10 minutes at 1200 x *g* with no brake, or acceleration. The top layer of monocytes separated by the dual gradient were removed and added to Red Blood Cell (RBC) lysis Buffer (1L–8.3g NH_4_Cl, 1g KHCO_3_, 0.04g Na_2_ EDTA 2H_2_O, 2.5g BSA) at a ratio of 1:3 and incubated for 3 minutes at room temperature with gentle mixing then spun at 400 x *g* for 6 minutes. The buffer was removed, and monocytes washed twice with 50 ml of PBS (Sigma-Aldrich), and PBS supplemented with Ca^+^ and Mg^++^ at 4 °C (Sigma-Aldrich).

Isolated monocytes were thereafter resuspended in 1 ml adhesion media (RPMI 1640 with L-glutamine; Thermo Fisher Scientific, supplemented with heat inactivated (56°C for 30 mins) 5% pooled human AB serum; Sigma-Aldrich, and 100 U/ml streptomycin, 100 U/ml penicillin; Sigma-Aldrich), counted on haemocytometer and then seeded into 48-well cell culture plates at a concentration of 1x10^6^ cells/well and incubated at 37°C at 5% CO_2_. The supernatant was removed 2 hours later, and replaced with differentiation media (adhesion media supplemented with 5% pooled human AB serum; Sigma-Aldrich and 20 ng/ml human M-CSF). Subsequent media replacements occurred on day 3 and day 6 post-isolation with adhesion media, and serum-free adhesion media, respectively. Human monocyte derived macrophages (HMDMs) were activated on day 7 with RPMI 1640 supplemented with 5% human AB serum (Sigma-Aldrich), 100 U/ml streptomycin, 100 U/ml penicillin, 0.5 μg/ml human IFN-γ; ImmunoTools; and 1 μg/ml *E*. *coli* LPS; Sigma-Aldrich) 24 hours before carrying out the phagocytosis assay.

### *Cryptococcus* infection of HMDMs

An overnight culture of the yeast was started by inoculating 3 mL of YPD (10 g/L yeast extract, 20 g/L bacteriological peptone, and 20 g/L glucose (Sigma-Aldrich) media, and incubated on a rotor at 20 rpm at 25 °C, prior to conducting the phagocytosis assay. In preparation for phagocytosis, yeast cells were washed in PBS, counted on a haemocytometer, and opsonized with 5% heat-inactivated human AB serum (Sigma-Aldrich). MDMs where then infected with 1x10^6^ yeast cells per well (MOI 10:1), and incubated at 37°C, 5% CO_2_ for 2 hours; after which non-engulfed yeast cells were washed away using PBS, and serum-free adhesion media was added to infected MDMs. At 0 (T0) and 18 (T18) hours post-infection, extracellular yeast cells were washed away using PBS and macrophages containing yeast cells were lysed in dH_2_O at 37 °C, 5% CO_2_ for 30 minutes. For live imaging to quantify vomocytosis, hMDMs were washed at T0, fresh serum-free RPMI added to infection wells and taken for imaging.

### CFU counts

Serial dilutions of the lysate from the phagocytosis assay were prepared and plated onto growth plates (2% YPD with 1% agar; Sigma-Aldrich) then incubated for 48 hours at 25°C. Intracellular proliferation rates were measured by dividing the number of counting colony-forming units per milliliter at T18 by those at T0.

### Live cell imaging

All time lapse images were captured on a Zeiss Axio Observer Live cell-imaging microscope enclosed within a humidified Okolab microscope chamber set at 37°C, 5% CO_2_, a Hamamatsu digital camera, LD Plan-Neofluar 20x/0.4 Korr Ph 2 M27 objective, 38 HE Green Flourescent reflector, using Zen software (Zeiss). 217 frames (1 frame, every 5 minutes for 18 hours) were taken from four different positions within each well to produce movies for manual analysis. Vomocytosis was measured as the percentage of intracellular cryptococci expelled from macrophages over the 18-hour period in all well positions.

### Cytokine profiling

Cell culture supernatants collected from donor MDMs at T0 and at T18 previously stored at -80 °C were thawed on ice in preparation for cytokine profiling. Initially, samples from 6 donors were probed for alternative or differential production of 27 human cytokines and chemokines using the Luminex Bio-plex (27-plex; Bio-Rad) which were then narrowed down to a panel of 7 cytokines that had previously been associated with cryptococcosis in HIV patients (IL-1β, IL-6, G-CSF, GM-CSF, IFN-γ, MCP-1 (MCAF), and TNF-α) due to inavailability of data in non HIV patients. We incorporated these cytokines into a custom-made Bio-plex Pro Human Cytokine 7-plex express assay for subsequent cytokine detection in 12 donors, in accordance with the manufacturer’s protocol. The fold change in cytokines released by donor MDMs was calculated by dividing the cytokine concentrations (pg/mL) at T18 by those at T0 for each donor. Correlation with matched vomocytosis and IPR data was carried out using the Graphpad Prism 6 software.

### Genotyping of small nucleotide polymorphisms

Consent for DNA analysis was obtained from 9 of the donors in this study. Genomic DNA was extracted from whole blood using Promega’s Wizard genomic DNA extraction kit. All primer sequences used to assess TLR2, Dectin-1 and ERK5 SNPs were designed by using the NCBI/ Primer-BLAST tool and are presented in S4 Table. The PCR products for each primer set were analyzed on a 1% agarose gel stained with Sybr Safe (Invivogen), and purified using Qiagen’s QIAquick PCR Purification Kit. For sequencing, we used the cycle sequencing technology provided by Eurofins Genomics on an ABI 3730XL sequencer. The results were analyzed by using 4peaks software, version 1.8; and multiple sequence alignments were generated using ClustalX, version 2.1.

## Results

**Fig 1:**
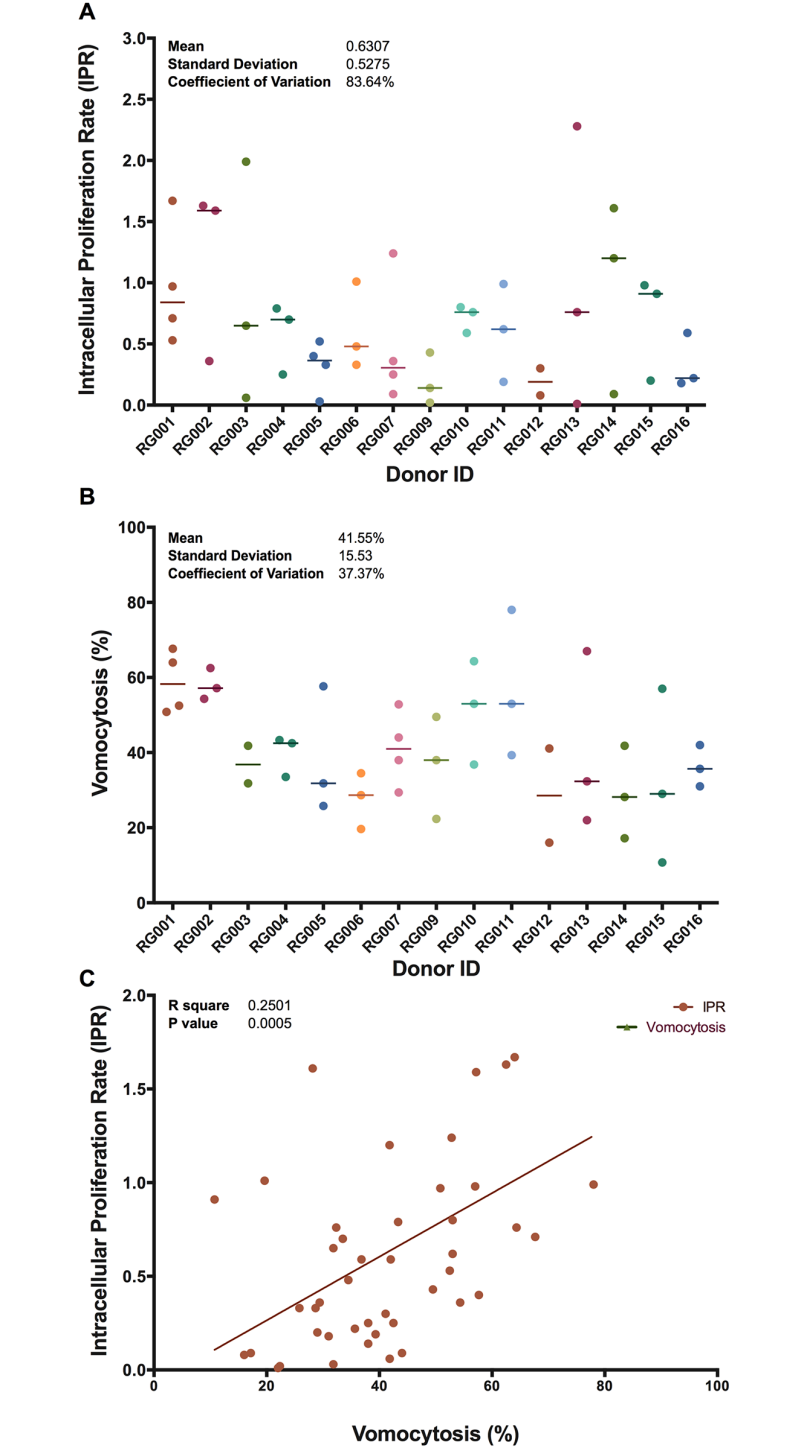
Variation in host responses to *C*. *neoformans* infections. (A) A measured intracellular proliferation rate (IPR) for each donor showing median of at least 2 biological repeats each (median = 0.53, mean = 0.6307, SD = 0.5275 and Coefficient of variation = 83.64%). (B) Variable rates of vomocytosis observed between and within donors showing median of at least 2 biological repeats each (median = 41.11%, mean = 41.55%, SD = 15.53 and Coefficient of variation = 37.37%). (C) Correlation between intracellular parasitism and non-lytic expulsion events (R square = 0.2501, P-value = 0.0005). Each point on the graph represents data from a single blood donation. Since not all participants were available to provide four repeat donations across the study period, the number of repeated samples varies from donor to donor.

To compare intra-donor and inter-donor variation in macrophage responses to infection with *C*. *neoformans*, we took repeated blood samples from a cohort of 15 healthy volunteers over a period of 2 years, taking into account seasonal effects on host immunity. We derived macrophages *in vitro* with macrophage colony-stimulating factor (M-CSF), and then analysed both intracellular fungal proliferation rates (IPR; Fig 1A) and the rate of non-lytic yeast expulsion from within macrophages (Fig 1B). Intracellular proliferation rate varied dramatically between and within donors (Fig 1A; S1 and S2 Tables) and consequently there was no significant difference in mean IPR between individual donors, suggesting that environmental variation (e.g. in the inflammatory status of the donor at the time of donation) is a more significant driver of variation in IPR than donor genetic background. In contrast, vomocytosis rate (calculated as the proportion of cryptococcal cells expelled from donor MDMs over an 18-hour period) was much less variable between repeat samples either within or between donors (p = 0.0820, one-way ANOVA; Fig 1B).

Interestingly, although vomocytosis shows much greater donor consistency than IPR, there is nonetheless a significant correlation between IPR and vomocytosis for individual samples (Fig 1C; R square = 0.2501, P-value = 0.0005).

Host cytokine profile has previously been shown to impact strongly on the response to cryptococcocal infection [23, 24]. We therefore wondered whether differences in the secreted cytokine and chemokine milieu during *in vitro* culture may influence their subsequent response to cryptococci. To test this, we quantified levels of seven cytokines and chemokines (interferon-γ (IFN-γ), TNF-α, interleukin-1b (IL-1b), interleukin-6 (IL-6), MCP-1, G-CSF and GM-CSF) in the media immediately following cryptococcal infection and again 18 hours later.

**Fig 2:**
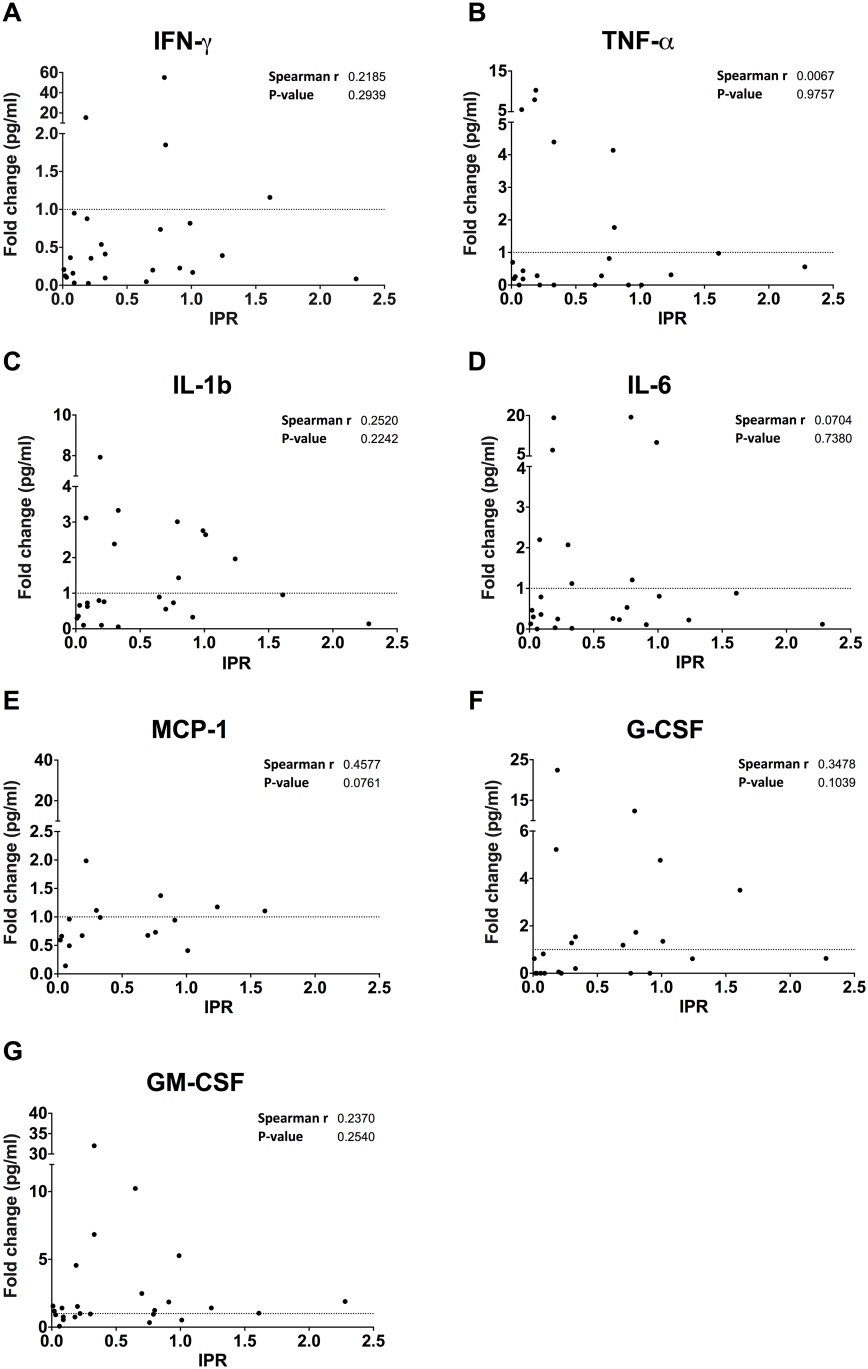
Correlation of fold changes in pro-inflammatory cytokines with IPR. Cytokines released over 18 hours were correlated with intracellular proliferation rates of KN99α-GFP from donor MDMs for 13 donors. Linear regression analysis revealed a significant association with e) MCP-1 (Spearman r = 0.4917; P-value = 0.0467); and no significant associations with a) IFN-γ; b) TNF-α; c) IL-1b; d) IL-6; f) G-CSF; nor g) GM-CSF. Note that only data points that crossed the detection threshold are shown hence not all graphs contain all data points. The fold change in cytokines released by donor MDMs was calculated by dividing the cytokine concentrations (pg/mL) at T18 by those at T0 for each donor.

**Fig 3:**
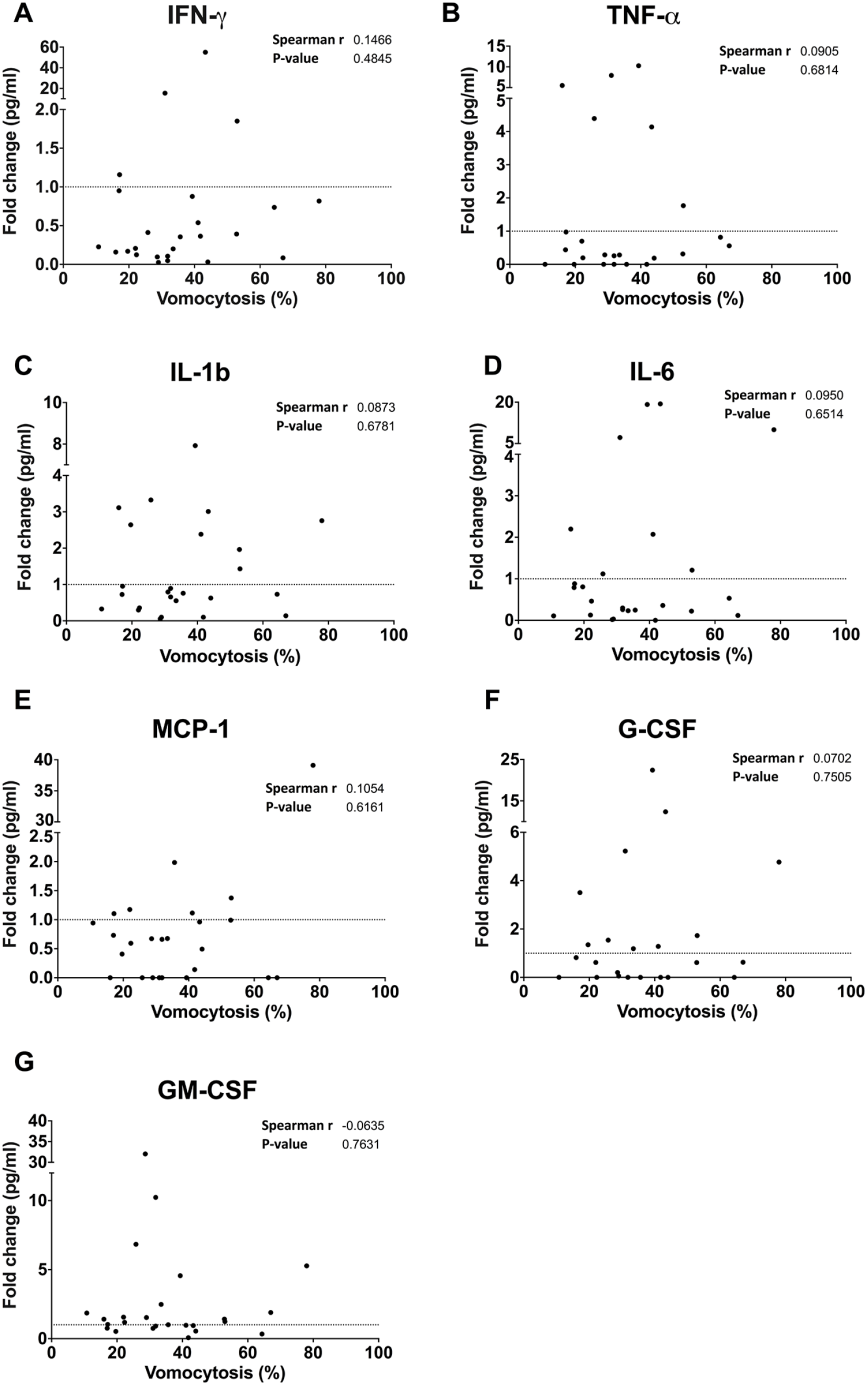
Correlation of pro-inflammatory cytokine profile from donor MDMs over 18-hours with non-lytic expulsion (vomocytosis) of KN99α-GFP. Linear regression analysis revealed no significant associations in 13 donors with a) IFN-γ; b) TNF-α; c) IL-1b; d) IL-6; e) MCP-1; f) G-CSF; nor g) GM-CSF. Note that only data points that crossed the detection threshold are shown hence not all graphs contain all data points. The fold change in cytokines released by donor MDMs was calculated by dividing the cytokine concentrations (pg/mL) at T18 by those at T0 for each donor.

We correlated them with measured IPRs (Fig 2) and vomocytosis rates for each donor (Fig 3 and S3 Table). Although cytokine levels varied dramatically between samples there was no significant correlation with either vomocytosis or IPR. Whilst MCP-1 showed a significant correlation with IPR (Spearman r = 0.4917; P-value = 0.0467), this result only just achieved significance and was primarily driven by a single outlier and should therefore be interpreted cautiously. Thus donor variation in autocrine inflammatory signaling during culture may contribute to, but cannot fully explain, the observed differences in cryptococcal response.

**Fig 4:**
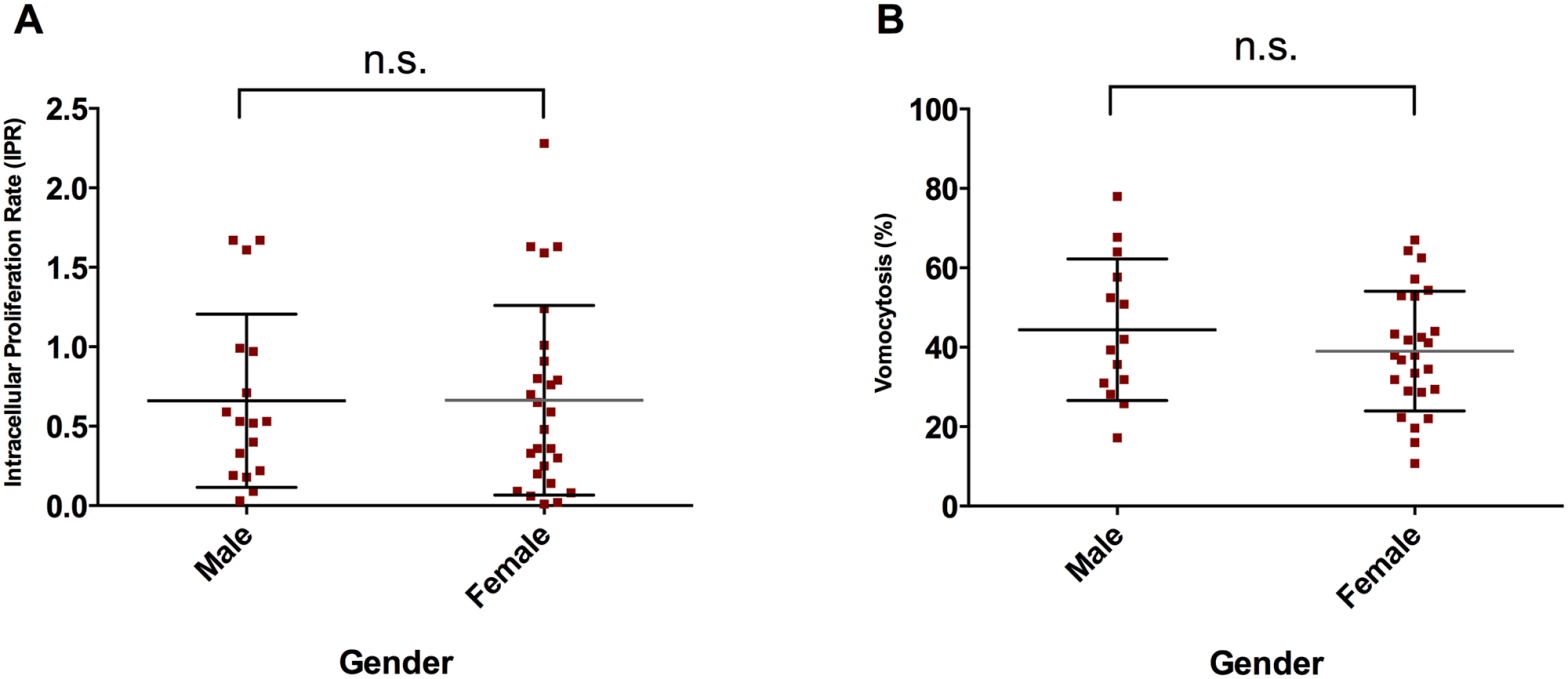
Analysis of gender contribution to observed variation. Two-tailed t-tests revealed no significant differences in a) intracellular virulence of KN99α-GFP (P-value = 0.9856); and b) non-lytic yeast expulsion from donor MDMs (P-value = 0.3181). Plot shows means, error bars are representative of standard deviations and n.s. states for non significant.

Given the established enhanced risk of cryptococcosis in men [25], we tested whether gender impacts on these macrophage responses *in vitro*. However, neither IPR nor vomocytosis rate showed variation with donor gender in this cohort (Fig 4).

Lastly, several immune signaling pathways have previously been shown to impact on vomocytosis/IPR [26]. In many cases, genes encoding key components of these pathways are known to be polymorphic in the human population and we therefore tested whether polymorphisms in three such genes (TLR2, dectin-1 or ERK5; S4 Table) may contribute towards the host variability that we report here. However, all of our tested donors carried the major allele for all of these polymorphisms, indicating that at least these three genes are unlikely to explain the detected variation in IPR and vomocytosis.

## Discussion

Previous attempts to characterize the differences in disease presentation and outcomes between HIV positive and negative CM patients suggest that exacerbated immune responses in otherwise healthy individuals contribute to the high mortality rates in non-HIV CM patients [27]. In this study, we aimed to establish the parameters of variation in “otherwise healthy” host macrophage interactions with *Cryptococcus neoformans*; placing a particular focus on the intracellular pathogenicity and vomocytosis of this pathogen. Even within the small cohort characterized here, it is clear that vomocytosis rates show significant intra-donor variation. In contrast, the ability of macrophages to control cryptococcal intracellular proliferation is highly variable even between samples from the same donor, suggesting that thus-far unidentified environmental factors impact strongly on this phenotype.

Levels of key inflammatory cytokines have been shown to impact strongly on cryptococcal disease progression in HIV patients [23]. However, the data we present here shows that varying cytokine profile does not explain the *in vitro* variation in vomocytosis or IPR. Thus the *in vivo* impact of cytokine profile on cryptococcosis most likely does not act at the level of single macrophage/fungus interactions.

Lastly, we found no evidence for variation being driven by polymorphisms in three key immune signaling genes: in TLR2 [28, 29], Dectin-1 [29, 30] and ERK5 [31, 32], suggesting that the underlying basis for the variation we detect either lies elsewhere in the genome or occurs at the level of environmental stimuli and not genetic polymorphism.

Ultimately, we hope that this study will help to define the parameters of the normal macrophage response to cryptococci and therefore prove useful both for subsequent analyses and for exploring disease heterogeneity in non-HIV cryptococcosis patients.

## Supporting information

S1 Fig Validation of GFP-tagged KN99α in comparison to wild-type KN99α strain. (A) GFP expressing strain shows no altered virulence in J774 macrophages with mean IPR (± Standard Deviation) for KN99α (2.938 ± 0.5953) and KN99α_GFP (3.084 ± 1.064). The GFP expressing strain also shows no altered response to the stress conditions B) NaCl; and C) H_2_O_2_; P-values for Wilcoxon test shown on graph.
(TIFF)

S1 Table Dates of blood donations for monocyte extraction from 15 study participants. (PDF)

S2 Table Intra-donor variation in monocyte-derived macrophage (MDM) responses to *C*. *neoformans* infections *in vitro*; means and medians shown are of at least 2 biological repeats per donor extrapolated from at least 2 technical repeats. (PDF)

S3 Table Correlation data for fold changes in detected cytokines with intracellular proliferation rate (IPR) and vomocytosis (%) of KN99α-GFP from MDMs from 13 healthy donors. (PDF)

S4 Table Details of small nucleotide polymorphisms and primers analyzed in 9 healthy donors. (PDF)
