# Supplementary figures and images for: Quantifying donor-to-donor variation in macrophage responses to the human fungal pathogen Cryptococcus neoformans
Source: PLoS One. 2018 Mar 29;13(3):e0194615. doi: 10.1371/journal.pone.0194615 (PMC5875765; doi:10.1371/journal.pone.0194615)

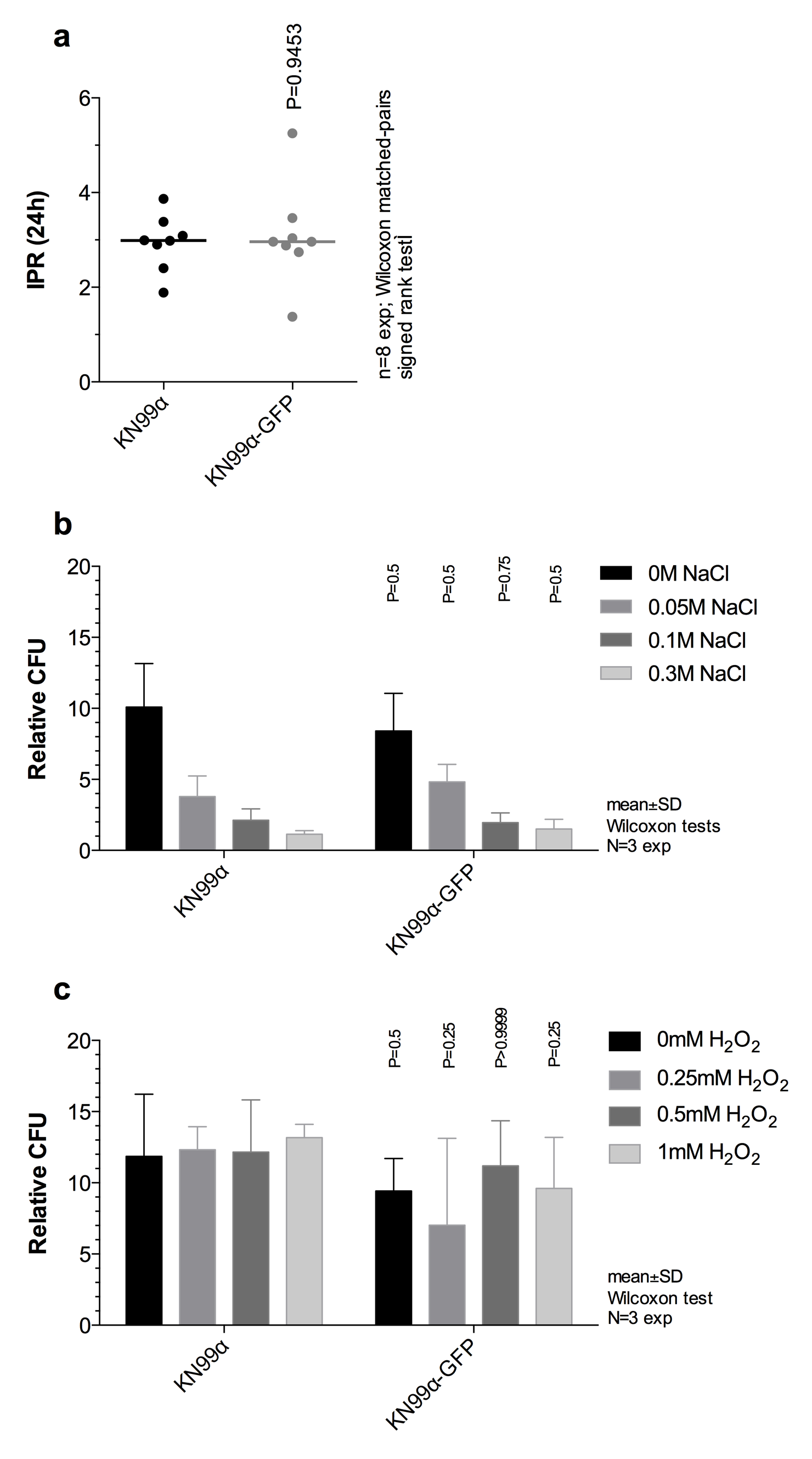

Supplement: S1 Fig — (A) GFP expressing strain shows no altered virulence in J774 macrophages with mean IPR (± Standard Deviation) for KN99α (2.938 ± 0.5953) and KN99α_GFP (3.084 ± 1.064). The GFP expressing strain also shows no altered response to the stress conditions B) NaCl; and C) H2O2; P-values for Wilcoxon test shown on graph. (TIFF) [file pone.0194615.s001.tiff]
