# Supplementary material for: Quantifying donor-to-donor variation in macrophage responses to the human fungal pathogen Cryptococcus neoformans
Source: PLoS One. 2018 Mar 29;13(3):e0194615. doi: 10.1371/journal.pone.0194615 (PMC5875765; doi:10.1371/journal.pone.0194615)
Supplement: S1 Table — (PDF) [file pone.0194615.s002.pdf]

Blood Donation Dates				
**Donor ID**	**N1**	**N2**	**N3**	**N4**
**RG001**	01.07.2015	21.07.2015	22.09.2015	03.02.2016
**RG002**	01.07.2015	21.07.2015	22.09.2015	
**RG003**	01.07.2015	21.07.2015	12.08.2015	
**RG004**	16.07.2015	12.08.2015	22.09.2015	
**RG005**	16.07.2015	20.01.2016	07.09.2016	22.05.2017
**RG006**	16.07.2015	20.01.2016	18.10.2016	
**RG007**	02.03.2016	20.04.2016	07.09.2016	09.08.2016
**RG009**	07.09.2016	18.10.2016	22.05.2017	
**RG010**	08.12.2016	03.02.2016	23.02.2016	
**RG011**	08.12.2016	12.01.2016	03.02.2016	
**RG012**	20.01.2016	23.02.2016		
**RG013**	09.02.2016	02.03.2016	16.08.2017	
**RG014**	09.02.2016	23.02.2016	09.08.2016	
**RG015**	03.02.2016	20.04.2016	09.08.2016	
**RG016**	20.04.2016	09.08.2016	18.10.2016	
