# Supplementary material for: Quantifying donor-to-donor variation in macrophage responses to the human fungal pathogen Cryptococcus neoformans
Source: PLoS One. 2018 Mar 29;13(3):e0194615. doi: 10.1371/journal.pone.0194615 (PMC5875765; doi:10.1371/journal.pone.0194615)
Supplement: S2 Table — (PDF) [file pone.0194615.s003.pdf]

Donor ID	Intracellular Proliferation Rate				Vomocytosis (%)			
	Mean	Median	Standard Deviation	Coefficient of variation	Mean	Median	Standard Deviation	Coefficient of variation
RG001	0.97	0.84	0.50	51.59%	58.75	58.25	8.35	14.20%
RG002	1.19	1.59	0.72	60.50%	58.00	57.17	4.15	7.15%
RG003	0.90	0.65	0.99	109.89%	36.83	36.83	7.07	19.20%
RG004	0.58	0.70	0.29	49.88%	39.78	42.50	5.45	13.71%
RG005	0.32	0.37	0.21	65.20%	38.43	31.83	16.93	44.05%
RG006	0.61	0.48	0.36	58.89%	27.60	28.67	7.49	27.15%
RG007	0.49	0.31	0.52	106.27%	41.06	41.00	9.87	24.05%
RG009	0.20	0.14	0.21	107.18%	36.61	38.00	13.64	37.25%
RG010	0.72	0.76	0.11	15.56%	51.39	53.00	13.82	26.90%
RG011	0.60	0.62	0.40	66.73%	56.78	53.00	19.61	34.54%
RG012	0.19	0.19	0.16	81.88%	28.56	28.56	17.76	62.18%
RG013	1.02	0.76	1.16	113.76%	40.44	32.33	23.57	58.28%
RG014	0.97	1.20	0.79	81.35%	29.05	28.17	12.34	42.49%
RG015	0.70	0.91	0.43	61.94%	32.25	29.00	23.30	72.23%
RG016	0.33	0.22	0.23	68.50%	36.22	35.67	5.52	15.24%
