# Supplementary material for: Quantifying donor-to-donor variation in macrophage responses to the human fungal pathogen Cryptococcus neoformans
Source: PLoS One. 2018 Mar 29;13(3):e0194615. doi: 10.1371/journal.pone.0194615 (PMC5875765; doi:10.1371/journal.pone.0194615)
Supplement: S3 Table — (PDF) [file pone.0194615.s004.pdf]

Cytokine/chemokine	IPR		Vomocytosis	
	Spearman r	P-value	Spearman r	P-value
**IL-1b**	0.2520	0.2242	0.0873	0.6781
**IL-5**	-0.2008	0.5015	-0.5989	0.0340
**IL-6**	0.0704	0.7380	0.0950	0.6514
**IL-17**	0.0332	0.9201	-0.3923	0.1795
**IFN-γ**	0.2185	0.2939	0.1466	0.4845
**TNF-α**	0.0067	0.9757	0.0905	0.6814
**G-CSF**	0.3478	0.1039	0.0702	0.7505
**GM-CSF**	0.2370	0.2540	-0.0635	0.7631
**MCP-1**	0.4917	0.0467	0.1054	0.6161
