# Supplementary material for: Quantifying donor-to-donor variation in macrophage responses to the human fungal pathogen Cryptococcus neoformans
Source: PLoS One. 2018 Mar 29;13(3):e0194615. doi: 10.1371/journal.pone.0194615 (PMC5875765; doi:10.1371/journal.pone.0194615)
Supplement: S4 Table — (PDF) [file pone.0194615.s005.pdf]

Pattern Recognition Receptor	SNP ID	SNP Details	Location	Global Minor Allele Frequency	Primer
**TLR2**	rs121917864	C>T missense	Intron	0.2849	Primer F<br>Primer R
**TLR2**	rs5743708	A>G missense	5' Near Gene	0.0068	Primer F<br>Primer R
**Dectin-1**	rs16910526	T>G missense	3' Near Gene	0.0409	Primer F<br>Primer R
**ERK5**	rs2233083	C>T Synonymous	Exon	0.0272	Primer F<br>Primer R
**ERK5**	rs3866958	G>T	Promoter	0.1837	Primer F<br>Primer R

Sequence
5'- CAGATGCTTTCTTCCCCTTTGAGA -3'<br>5'- CGGAAATGGGAGAAGTCCAGT -3'
5'- TACAGTGAGCGGGATGCCT -3'<br>5'- TATCGCAGCTCTCAGATTTACCC -3'
5'- TGCAGCTAGTAGCAGTTCTTG -3'<br>5'-CTCCACCCTTCCTCTTACATTGA -3'
5'- GTTCTCAGGCACACCAAAGG -3'<br>5'- AAGGATTGTTCAGGGGGAGTTG -3'
5'- CGAACCCTCCACTGACTTCC -3'<br>5'- CCTTTCCTTCCAGCTCACAGT -3'
